# Supplementary material for: Patterns of Intron Gain and Loss in Fungi
Source: PLoS Biol. 2004 Nov 30;2(12):e422. doi: 10.1371/journal.pbio.0020422 (PMC532390; doi:10.1371/journal.pbio.0020422)
Supplement: Table S1 — Also available at http://genes.mit.edu/NielsenEtAl/. (4.3 MB ZIP). [file pbio.0020422.st001.zip › NielsenEtAl/html/160.html]

AN9470.1.NCU07853.1.MG05283.1.FG04126.1


```
 CLUSTAL W (1.82) Multiple Sequence Alignments - Introns Inserted


Sequence 1: AN9470.1	301 aa
Sequence 2: FG04126.1	302 aa
Sequence 3: NCU07853.1	295 aa
Sequence 4: MG05283.1	263 aa
Alignment Length: 302 aa
Number Identitical Residues: 146 aa
Alignment Score (without introns) 7509


MG05283.1 	--------------------------------MSPTGPLTRYDEHS~FTKADNSVVVATD
NCU07853.1	MARLSAARYGKDNVRVYKVHKDEKTGQQSVTEMTVCCLLEGEIDTS~YTQADNSVIVATD
FG04126.1 	MPYVSAARYGKDNVRVCKVDRDSSTGVQTVTEMTVCCLLEGEIETS~YTQADNSVVVATD
AN9470.1  	MSTVAAARYGKDNVRVYKVHKDPKTGVQTVTEMTVCVLLEGEIDTS2YTKADNSVIVATD
          	 .  :::  ....    .   . .:. .: :.*:    *    : * :*:*****:****

MG05283.1 	SIKNTIYIKARETPVNPPELYASILGQHFIDTYPHIHTAHVDVIVHRWTRMTIDGQPHPH
NCU07853.1	SIKNTIYIKAKEHPVNPPELYASILGQHFLDKYAHISAAHIDVTVHRWTRMVIDGQPHPH
FG04126.1 	SIKNTIYITAKENPVNPPELYASILGSHFIEKYKHIHVANVSVKTVRWARLDVDGKPHPH
AN9470.1  	SIKNTIFILAKQNPVTPPELFGSILGTHFINKYKHIHVAHTNIITHRWTRLNIDGKPHSH
          	******:* *:: **.****:.**** **::.* ** .*: .: . **:*: :**:**.*

MG05283.1 	SFFRDGQETRTVGAVVRRGSGLALRSGIAGLSVLKSTGSAFHGFVRDEFTTLPETWDRIL
NCU07853.1	SFLRDGQETRNVEATVTR-DGITIKSGIVGLQVLKSTGSAFHGFVRDEFTTLKETWDRIL
FG04126.1 	SFFKDGEETRNVEVRVSRQEGIEIKSSLVGLTVLKSTGSAFHGFVRDEYTTLPETWDRIF
AN9470.1  	SFVRDSEETRNVQVDVTEGVGIDIKSSINKLTVLKSTGSQFWGFVRDEYTTLPEVWDRIL
          	**.:*.:***.* . * .  *: ::*.:  * ******* * ******:*** *.****:

MG05283.1 	STDVDCGWSWKSFPDLDAVKAIVPKFDQAWEDARSITMKTFATDDSASVQATMYKMCEQI
NCU07853.1	STDVDAGWTWKKCANLAAVQDGVERFDAAWEAARNITLKTFAEDESASVQNTMYKMCEQI
FG04126.1 	STDVDATWKWKKFDSVDAVKQFAPKFDTVREAARNITLKTFAEDASASVQATMYKMSDLI
AN9470.1  	STDVEATWAWKRFSGLDEVRGNVPKFDETWEAARNITLKTFAEEESASVQATMYKMGEQI
          	****:. * **   .:  *:  . :** . * **.**:**** : ***** ***** : *

MG05283.1 	LEAAPEVEKTDYSLPNKHYFEVD1LSWHKGLANTGKDAEVYVPQSGPNGLIKCEVSR---
NCU07853.1	LDAVPETEKTNYSLPNKHYFEVD1LSWHKGLQNTGKDAEVYAPQSGPNGLIKCEVSRS--
FG04126.1 	LESVPEVATVTYSLPNKHYFEID1LSWHKGIKNTGKDAEVYAPQSGPNGLIKCEVSRDSL
AN9470.1  	LAYQPLLETVEYSLPNKHYFEID1LSWHKGLKNTGKDAEVFVPQTNPNGLIKCTVGRKS-
          	*   *   .. **********:* ******: ********:.**:.******* *.*.: 

MG05283.1 	----
NCU07853.1	----
FG04126.1 	QSKL
AN9470.1  	KAKL
          	.:.
```
